# Carbonic Anhydrase as a Biomarker of Global and Local Impacts: Insights from Calcifying Animals

**DOI:** 10.3390/ijms20123092

**Published:** 2019-06-25

**Authors:** Yuri Dornelles Zebral, Juliana da Silva Fonseca, Joseane Aparecida Marques, Adalto Bianchini

**Affiliations:** 1 Programa de Pós-Graduação em Ciências Fisiológicas, Instituto de Ciências Biológicas, Universidade Federal do Rio Grande, Avenida Itália km 8, Rio Grande 96203-900 RS, Brazil; yurizebral@gmail.com (Y.D.Z.); julianafonseca94@hotmail.com (J.d.S.F.); 2 Programa de Pós-Graduação em Oceanografia Biológica, Instituto de Oceanografia, Universidade Federal do Rio Grande, Avenida Itália km 8, Rio Grande 96203-900 RS, Brazil; jmarques.oceano@gmail.com; 3 Instituto Coral Vivo, Rua dos Coqueiros, Parque Yaya, Santa Cruz Cabrália 45807-000 BA, Brazil

**Keywords:** temperature, warming, acidification, calcification, Contaminant, pollution, bioindicator, ecotoxicology, environmental assessment

## Abstract

The emission of greenhouse gases has grown in unprecedented levels since the beginning of the industrial era. As a result, global climate changes, such as heightened global temperature and ocean acidification, are expected to negatively impact populations. Similarly, industrial and urban unsustainable development are also expected to impose local impacts of their own, such as environmental pollution with organic and inorganic chemicals. As an answer, biomarkers can be used in environmental programs to assess these impacts. These tools are based in the quantification of biochemical and cellular responses of target species that are known to respond in a sensitive and specific way to such stresses. In this context, carbonic anhydrase has shown to be a promising biomarker candidate for the assessment of global and local impacts in biomonitoring programs, especially in marine zones, such as coral reefs, considering the pivotal role of this enzyme in the calcification process. Therefore, the aim of this review is to show the recent advances in the carbonic anhydrase research and the reasons why it can be considered as a promising biomarker to be used for calcifying organisms.

## 1. Carbonic Anhydrase and Its Biological Function

### 1.1. Carbonic Anhydrase

**Figure 1:**
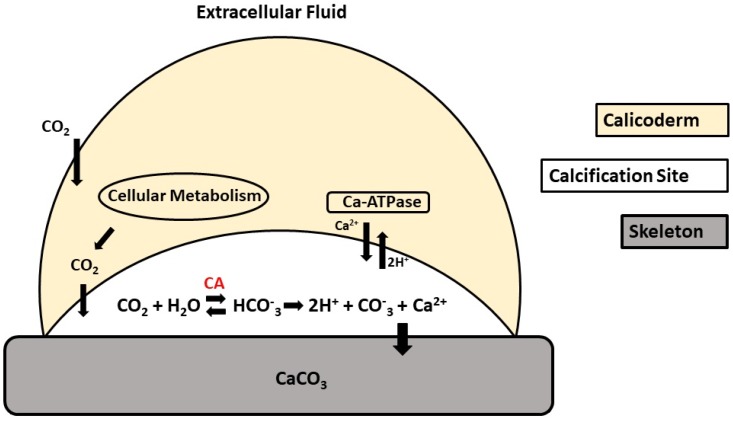
Schematic representation of the calcification process in scleractinian corals. Adapted from Zilberberg et al. (2016) [39].

Carbonic anhydrase is a zinc metalloenzyme found in animals, plants, algae, and prokaryotes. This enzyme catalyzes the hydration reaction of carbon dioxide (CO_2_) into bicarbonate (HCO_3_^−^) and protons (H^+^), playing a crucial role in different physiological processes. Five carbonic anhydrase families, referred to as α, β, γ, δ, and ζ have been identified in animals and plants. Among their numerous functions, carbonic anhydrase (particularly the α family) are known to play a key role in biocalcification processes, i.e., the ability to deposit calcium carbonate crystallites in a controlled manner [1,2] (Figure 1). The α-carbonic anhydrase has many isoforms that differ by their enzymatic activity, kinetic properties, sensitivity to inhibitors, and tissue and subcellular distribution. α-carbonic anhydrase can be cytosolic, mitochondrial, membrane-bounded, or secreted. The structure of the α-carbonic anhydrase family is characterized by three histidine residues that bind the zinc cofactor, and a proton shuttling residue at the entrance of the active site is responsible for converting a zinc-bound water molecule to a hydroxide ion. Besides these, gatekeeper residues (Glu106 and Thr199, human carbonic anhydrase) allow the optimal orientation of the zinc-bound hydroxide ion to enhance the nucleophilic attack of the substrate (CO_2_) [3,4].

### 1.2. Biological Function

In animals, carbonic anhydrase is present in many different tissues and is involved in numerous physiological processes, including osmoregulation, ion transport, acid–base regulation, respiration, and calcification. In algae and plants, this enzyme plays an important role in photosynthesis [5].

Osmoregulation is the ability of some organisms to actively maintain osmotic concentrations in extracellular fluids despite the surrounding environmental salinity [6]. In aquatic animals, the gills play an essential role in this process as this tissue is the main organ involved in ion exchange and ventilation [7,8]. Aquatic organisms have high concentrations of carbonic anhydrase in this tissue, where this enzyme plays an essential role in maintaining constant levels of osmolytes, such as Na^+^ and Cl^−^, that are important for cell function. For this, the basolateral Na^+^/K^+^-ATPase provides the electrochemical force for Na^+^ and Cl^−^ uptake, whereas the branchial cytosolic carbonic anhydrase provides the demanded counterions H^+^ and HCO_3_^−^. Interestingly, the process of ionic regulation is coupled with acid–base balance because the regulation of ions relies primarily on the direct transfer of H^+^ and HCO_3_^−^ across the gill in exchange for Na^+^ and Cl^−^, respectively [5,9]. In addition to gills, the intestine is also an important site for Na^+^ and Cl^−^ transport in order to maintain osmotic balance in marine teleosts. Absorption of Cl^−^ occurs in part via Cl^−^/HCO_3_^−^ exchange, with HCO_3_^−^ provided by carbonic anhydrase [10,11]. Hydration of CO_2_ also liberates H^+^, which is eliminated mainly across the basolateral membrane of the intestinal epithelium with Na^+^ reabsorption [12,13,14].

Another important function of carbonic anhydrase is involved with excretion of metabolites. Branchial carbonic anhydrase localization in a subcellular compartment that is accessible to hemolymph or plasma HCO_3_^−^ can function in CO_2_ excretion [15,16]. Also, branchial carbonic anhydrase is responsible for acidification of the apical membrane via a H^+^-ATPase and Na^+^/H^+^-exchanger facilitating the excretion of ammonia [17]. Interestingly, other tissues involved in both respiration and ion transport are also known to possess membrane-associated carbonic anhydrase. For example, the mammalian lung has carbonic anhydrase associated with the endothelial membrane that is exposed to plasma [18], and the kidney tubule also has carbonic anhydrase present on the luminal brush borders [19].

Carbonic anhydrase is also important for mitochondrial metabolism as the HCO_3_^−^ resulting from this enzyme activity provides an intra mitochondrial counter-anion facilitating Ca^2+^ accumulation in the mitochondrial matrix in the form of CaCO_3_ [20]. Another important role of carbonic anhydrase in energetic metabolism is to provide HCO_3_^−^ for pyruvate carboxylation into oxaloacetate. Following its synthesis, this molecule is decarboxylated and simultaneously phosphorylated to produce phosphoenolpyruvate in the cytosol or in the mitochondria and then is transported out of the mitochondrial matrix to be converted into glucose in cytosolic gluconeogenic processes [21].

Carbonic anhydrase plays an important role in biological calcification, a fundamental process performed by a great number of marine invertebrates. This process occurs at the interface between organic and mineral tissues, with the formation of an organic matrix that acts as the deposition center of CaCO_3_ crystals [22,23], termed as the calcification site [24]. At this location, carbonic anhydrase catalyzes the reversible reaction of CO_2_ hydration, culminating in the formation of HCO_3_^−^ and H^+^, a fundamental process for the supply of dissolved inorganic carbon for skeleton synthesis.

In association with carbonic anhydrase, Ca-ATPase participates in the calcification process by delivering Ca^2+^ into the calcification site concomitantly with H^+^ removal, directing the calcification reaction (Ca^2+^ + CO_2_ + H_2_O ↔ CaCO_3_ + 2H^+^) toward the formation of CaCO_3_ [25]. By removing H^+^, Ca^2+^-ATPase maintains an alkaline pH (>8.20) at the calcification site [26,27], improving the passive diffusion of CO_2_ and increasing the efficiency of the calcification process [28] (Figure 1).

The role of carbonic anhydrase in the calcification process has been studied mainly in scleractinian corals; however, similar mechanisms have already been described in mollusks [2,29,30,31], sponges [32], octocorals [33], hydrocorals [34], gorgonian [35], crabs [36,37], algae [38], and foraminifera [39], exemplifying the essential role of carbonic anhydrase in the regulation of CaCO_3_ precipitation in various biological systems.

## 2. Untangling Global and Local Impacts

### 2.1. Global Impacts

Global impacts can be defined as environmental changes that negatively affect a substantial part of the globe. The most important examples of global impacts are those caused by global climate change, such as the loss of ice mass in the cryosphere, sea level elevation, ocean acidification, and global warning [40]. The main mechanism driving global changes is the elevated emission of greenhouse gases, such as perfluorocarbons, sulfur hexafluoride, hydrofluorocarbons, CO_2_, methane, and nitrous oxide. These gases are responsible for the expansion of Earth’s greenhouse effect and consequent heating [41]. Among these gases, CO_2_, methane, and nitrous oxide are pointed to as the major drives of global change [42].

Within the context of global impacts, ocean acidification is expected to impose major impacts in both biological and economical systems [40]. This process occurs when the excessive amount of anthropogenically derived CO_2_ gets dissolved into seawater and disrupts the fine-tuned equilibrium of inorganic carbon in the ocean, culminating in less carbonate ions (CO_3_^2−^) and reduced calcium carbonate saturation state (ΩCc) in favor of elevated levels of protons (H^+^) and bicarbonate ions (HCO_3_^−^). Resulting from this, a reduction of 0.3–0.5 pH units is expected for the seawater surface by the end of this century [40]. The ocean acidification process can profoundly impact reefs around the world as corals and other calcifying organisms greatly depend on elevated seawater pH in order to synthetize carbonate skeletons, structures responsible for the well-known tridimensional shape of this ecosystem [40]. Moreover, reductions in ocean pH can also impact marine species in terms of survival, growth, development, and abundance [43].

In a close relationship with ocean acidification, global warming is another example of the impact resulting from anthropogenic activities [44,45]. Actually, global mean temperature is expected to rise by 2.6–4.8 °C by the end of 21st century [40]. This fact has been causing deep concerns in the scientific community considering that temperature is a major driver of physiological functioning; therefore, global warming is expected to greatly impact biological systems around the world [40,46]. For example, it is known that elevated temperature can lead to an unproportioned sex rate [47,48], induce disturbances on energetic metabolism [49,50], alter the antioxidant system [51,52,53], elevate cellular damage [54], and elevate oxidative stress and oxidative damage [53,55,56].

### 2.2. Local Impacts

As opposed to global impacts, local impacts are defined as environmental stresses that negatively affect organisms and populations at a narrow geographic scale [40,57], such as specific rivers and lakes, coastal areas (such as river mouths, estuaries, and beaches), and grass plains and forests. These impacts can present themselves in the most diverse ways; notwithstanding, they are usually caused by anthropogenic activities [40]. For example, poor land-use and watershed management can lead to impacts related to harbor and mining activities, impacts related to large-scale agricultural and cattle production systems, and to impacts related to sewage and industrial waste [58]. It is important to note that the majority of these negative effects are related to the contamination of aquatic environments, and therefore, the loss of water quality in natural areas is considered to be a major result of local impacts [40,58].

The contaminants that usually reach aquatic environments can be classified as organic or inorganic molecules/elements [59]. Organic contaminants are composed primarily of carbon, hydrogen, and possibly other elements, such as nitrogen and phosphorus. Many of these molecules are synthetized and used by humans for economic purposes, such as herbicides, biocides, pharmaceuticals, solvents, chemical precursors, and fossil fuels [60]. Nonetheless, there are also organic contaminants that are produced in natural processes, such as microcystins derived from harmful algal blooms [61]. On the other hand, inorganic contaminants include elements, such as metals and salts, that are usually found in dissolved forms, like cations and anions. In accordance with organic contaminants, these elements are usually derived from anthropogenic processes such as mining and harbor activities or from sewage and industrial waste [62].

### 2.3. Tangled Effects: The Role of Interactions

The literature describing negative effects of local and global impacts are majorly based in single-factored laboratory studies; however, these impacts are occurring together, which can lead to unexpected combined effects and even complex interactions [59,63]. The nature of this combination may be expressed in diverse ways. For example, it can succeed as the combined effects being equal to the sum of the individual effects, which is called an additive effect. On the other hand, factor–factor interactions may lead to combined effects that are smaller than the sum of single effects, termed antagonist effects. Finally, interactions may lead to combined effects that are greater than the sum of individual effects, which are called synergistic or potentiated effect [64]. This last outcome raises concerns regarding local and global impacts, as environmental laws and conservational programs may be based in scientific findings that do not consider the role of combined effects and that are possibly underestimating true impacts [65]. As an answer, the use of more complex, multi-factorial experiments is required for a more precise assessment. Similarly, it is also important to assess biomarkers in bioindicator species that are responsive to such interactive effects. With that in mind, the aim of this review is to show why carbonic anhydrase can be considered as a good biomarker to be used in calcifying animals in order to assess global and local impacts and the possible interactions between them.

## 3. Bioindicators and Biomarkers

### 3.1. Bioindicators

Bioindicators can be defined as species used to evaluate biotic and abiotic environmental characteristics throughout a short or a long period. These biological tools have been used for different purposes, such as estimating biological diversity [66], assessing ecological characteristics [67,68,69], and evaluating the outcomes of environmental impacts [70,71]. Animals have been used as indicators for a long time; for example, at the beginning of the 20th century, birds were introduced in coal mines as bioindicators of lethal levels of carbon monoxide [72]. Similarly, cats helped to discover the Minamata disease, a syndrome caused by a severe mercury poisoning that occurred at the early 1900s in Japan [73].

To be considered as good bioindicators, species need to be sensitive to environmental changes, have to be easy to collect, and have a well-known taxonomy, ecology, and geographical distribution [74]. The main advantage of using bioindicators to evaluate environmental impacts rather than just using the direct quantification of physical or chemical parameters is that the response from animals to environmental stress is given as a time-integrated endpoint [75], reducing the sampling frequency and elevating the explanatory power. Within this context, focal species can be used as bioindicators. The concept behind this is to use the tolerance limit of sensitive organisms as the environmental threshold in conservation laws, aiming to protect other species and ecosystems [76]. In the context of biomonitoring programs, endpoints of many levels of biological organization are assessed in bioindicators species, such as cellular, biochemical, physiological, histological, and organismal levels. These endpoints are coined as biomarkers [6].

### 3.2. Biomarkers

Biomarkers are endpoints measured in bioindicator species that respond to environmental changes in a well-known manner, and therefore, can be used to identify and track environmental impacts [6]. These endpoints can be the expression of genes, the activity of enzymes, the concentration of proteins, growth, behavioral patterns, reproductive success, and many other biological processes [6,77,78]. Despite that, as just mentioned, these endpoints are assessed at different levels of biological organization, and interestingly, the behavior of these biomarkers are deeply structured within these different levels [78]. For example, biomarkers related to gene expression and enzymatic activity are the fastest responding endpoints and reflect environmental changes that occur over a short period. On the other hand, biomarkers that are assessed at the organismal level, such as growth, behavior, and reproduction, involve a longer time scale and can be used in studies that aim to assess environmental impacts over the long term. In the middle of these two, endpoints related to the quantification of macromolecules produced by organisms, such as proteins and steroid hormones, are biomarkers that respond over an intermediate time scale and can help to stablish mechanistic links between the impacts at lower and higher biological scales [6,77,78].

At this point, it is important to comment that biomonitoring programs can be based in the quantification of a single biomarker or multiple biomarkers from the same level of biological organization; nevertheless, the explanatory potential of these programs can be greatly enhanced if the endpoints evaluated are from different hierarchical levels. For example, organismal biomarkers reflect chronic populational impacts that are occurring at the present moment; on the other hand, molecular and biochemical biomarkers can be used to predict populational impacts yet to come. In this way, it is possible to estimate on-going environmental issues but also to predict, and possibly avoid, future impacts before they reach populational or ecosystem scales [77,78].

## 4. Carbonic Anhydrase as a Biomarker in Calcifying Organisms

### 4.1. Global Impacts: Effects of Water Acidification

Ocean acidification alters carbonate chemistry, deeply affecting the process of biological calcification. The process of calcification can be observed in a wide variety of organisms, from protists (e.g., foraminifera) to algae (e.g., coralline algae) and animals (e.g., corals and bivalves) [79,80,81], suggesting that sea water acidification pose a high risk to aquatic species abundance and richness. Since coral reefs are one of the most valued marine ecosystems, and its characteristic tridimensional shape is built and maintained basically by calcifying organisms, the negative impacts of ocean acidification on reef-building species have become a major concern of the scientific community [82,83,84]. 

Indeed, many studies have already described how a considerable number of calcifying species respond to ocean acidification in terms of carbonic anhydrase gene transcription. In most cases, pH reduction leads to a down-regulation in these genes’ transcription; therefore, there is enough evidence to consider this endpoint as a suitable biomarker. Despite that, most of the studies were performed with coral species. For example, the larval form of the coral *Acropora millepora* had a down-regulation in the membrane-bounded carbonic anhydrase gene following exposure to CO_2_-driven water acidification (pH 7.96 and 7.86) for 3 days [85]. Moreover, Zoccola et al. (2016) [86] demonstrated that the coral *Stylophora pistillata* had a down-regulation in two isoforms of carbonic anhydrase genes following long-term (1 year) exposure to a pH of 7.20. Similar results have also been found in calcifying organisms other than corals; for example, Richier et al. (2011) [87] showed that water acidification (pH 7.85) for 8 days reduced the expression of the α and γ carbonic anhydrase isoforms in the coccolithophore *Emiliania huxleyi*. Also, the oyster *Crassostrea gigas* exposed to saltwater at pH 7.50 for 16 days showed a down-regulation in the α carbonic anhydrase gene transcription in diverse tissues [88]. On the other hand, a few studies showed that ocean acidification may induce up-regulation in carbonic anhydrase genes. For example, Carreiro-Silva et al. (2014) [89] demonstrated that the α isoform of this gene was up-regulated when the cold-water coral *Desmophyllum dianthus* was exposed to water acidification (pH 7.70) over 8 months. Similarly, Vidal-Dupiol et al. (2013) [90] showed that when the coral *Pocillopora damicornis* was exposed to pH values of 7.80 and 7.40, the transcription of carbonic anhydrase genes was up-regulated (both extracellular and cytosolic isoforms). The reason for this incongruency is unclear considering that it is not related to the period to which animals were exposed to water acidification and/or its intensity, and it also does not seem to be phylogenetically structured (more similar in close related species); therefore, this divergence actually appears to be specific to a few species. With that in mind, it is suggested that in order to be used as a reliable biomarker of ocean acidification, the patterns of gene expression of carbonic anhydrase isoforms have to be previously evaluated under controlled conditions in order to evaluate whether this endpoint will be up- or down-regulated in the target species.

The influence of ocean acidification in the carbonic anhydrase activity of calcifying organisms has also been extensively studied. As the reader will see, this endpoint responds to reductions in water pH with remarkable reproducibility, even despite possible confounding factors, such as phylogeny, acidification intensity, and exposure duration. Interestingly, calcifying organisms respond to water acidification, in terms of carbonic anhydrase activity, in a similar manner to what was described for the gene transcription of this protein, that is, the activity of this enzyme was reduced as water pH was lowered. Even more interestingly, most of the studies describing this process was conducted with bivalve mollusks, despite what was observed in the case of carbonic anhydrase gene transcription, which has been majorly studied in coral species. For example, this enzyme activity was inhibited in the oyster *C. gigas* exposed to water at pH 7.50 for 16 days [88]. Similarly, the branchial carbonic anhydrase activity in the oyster *Crassostrea virginica* exposed to water acidification (pH 7.95) for 2 weeks was also reduced. Moreover, low pH levels (6.50, 7.10, and 7.70) also inhibited this enzyme’s activity in the mussel *Mytilus edulis* following 14 and 21 days of exposure [91]. In accordance, the independent work by Fitzer et al. (2014) [92] also showed that M. edulis exposed to water acidification at different intensities (pH levels of 7.70, 7.50, 7.40, 7.30, and 7.20) for 1 month suffered inhibition of the carbonic anhydrase activity in the mantle and extrapallial fluid. Similarly, Moreira et al. (2016) [93] demonstrated that two oyster species from *Crassostrea* genus (*C. gigas* and *C. angulata*) showed reduced activity of this enzyme when exposed to acidified water (pH 7.30) for 28 days. The influence of water acidification in the carbonic anhydrase activity has also been evaluated in coral species. For example, Marangoni et al. (2019) [83] showed that the zooxanthellate scleractinian coral *Mussismilia harttii* experienced a reduction in this enzyme activity following exposure to acidified water (pH levels of 7.50 and 7.20) for 15 and 35 days. Similarly, when the coral *S. pistillata* was cultivated for 1 year in low-pH water (pH 7.20), a reduction in carbonic anhydrase activity was also observed [86]. In contrast, Marangoni et al. (2017) [34] demonstrated that the calcareous hydrozoan *Millepora alcicornis* elevated the activity of this enzyme following exposure to extreme water acidification (pH 7.20) for 30 days.

### 4.2. Global Impacts: Effects of Warming

Global warming is expected to greatly impact calcifying organisms, and therefore, some studies have aimed to understand to what extent the elevation of water temperature could negatively affect these animals. However, the literature describing the impacts of heated water in carbonic anhydrase activity and gene transcription are not as prolific as it was seen in the case of ocean acidification. Even so, there is sufficient information to draw a major picture of this effect. For example, Edge et al. (2005) [94] showed that the coral *Montastraea faveolata* had a reduced transcription in the carbonic anhydrase gene following temperature elevations of 4 and 7 °C for 4 h. Similarly, Ogawa et al. (2013) [95] demonstrated that the gene transcription of a cytosolic isoform of this protein was down-regulated when the coral *Acropora aspera* was exposed to a temperature elevation of approximately 5 °C for 2 weeks. Moreover, the same study showed that this coral species also had a down-regulation in the membrane-bounded isoform of carbonic anhydrase gene following heat stress for 1 day. On the other hand, Hoadley et al. (2015) [96] demonstrated that the coral *Acropora millepora* exposed for 24 days to a temperature elevation of 5 °C raised the gene transcription of an intracellular isoform of this protein; conversely, the extra-cellular isoform of carbonic anhydrase was unresponsive to heat treatment in this species. Similarly, the same study showed that the coral *P. damicornis* was also unresponsive to water temperature elevation in terms of the gene transcription of both carbonic anhydrase isoforms. 

Moving forward, the effects of water temperature elevation in the carbonic anhydrase activity has also been evaluated in some calcifying organisms, although by a reduced number of studies, considering that only two works aimed to evaluate the sole role of heat stress in the activity of this enzyme. First, Ivanina et al. (2013) [97] showed that the oyster *C. virginica* exposed to heat stress (5 °C elevation) for 2 weeks elevated the carbonic anhydrase activity in the mantle edge, and following 8 weeks, the activity of this enzyme was augmented in the gills. Similarly, Ivanina et al. (2013) [97] also demonstrated that the clam *Mercenaria mercenaria* exposed to the same experimental conditions for 8 weeks showed elevated enzymatic activity in the mantle edge, and following heat stress for 15 weeks, carbonic anhydrase activity was augmented in both tissues (mantle edge and gills). Following Ivanina’s work, Fonseca et al. (2017) [52] showed that heat stress (1.6 and 2.3 °C elevation) for 8 and 12 days induced an elevation in carbonic anhydrase activity in the coral *M. harttii*.

### 4.3. Local Impacts: Effects of Environmental Pollution

Within the context of local impacts, the process of environmental contamination has been raising deep concerns in the scientific community [98]. As a reflection of that, a great number of studies have aimed to understand how pollutants could affect the carbonic anhydrase activity and its applicability as a pollution biomarker. As the reader will see, the majority of these studies showed that this enzyme activity can be inhibited by a multitude of contaminants. For example, El-Gendy et al. (2019) [99] demonstrated that the land snail *Theba pisana* orally exposed to three organic contaminants (abamectin, thiamethoxam, and acrylamide) for 2 weeks displayed reduced carbonic anhydrase activity. Similarly, Lionetto et al. (2006) [78] demonstrated that the mussel *Mytilus galloprovincialis* exposed to cadmium chloride, both in vitro and in vivo (incubation for 1 h or exposure for 14 days, respectively), had a remarkable inhibition in carbonic anhydrase activity. Similarly, the oysters *C. giga* and *C. angulata* also showed reduced activity of this enzyme when exposed to 2.78 mg/L arsenic for 28 days [93]. Moreover, Santini et al. (2011) [100] showed that the freshwater bivalve *Anodonta anatina* exposed to low levels of copper for 15 days displayed reduced carbonic anhydrase activity. Similarly, this enzyme was inhibited when the corals *Acropora cervicornis* and *M. faveolata* were exposed to this metal for 5 weeks [101]. Additionally, Marangoni et al. (2019) [83] showed that the coral *M. harttii* exposed for up to 35 days to 2.3 or 3.2 μg/L copper also experienced inhibition of this enzyme. Similarly, Fonseca et al. (2019) [102] demonstrated that the same coral species exposed to many copper concentrations (4.6–19.4 μg/L) for 96 h displayed reduced carbonic anhydrase activity. In opposition, Caricato et al. (2010) [103] showed that the mussel *M. galloprovincialis* exposed to cadmium elevated the carbonic anhydrase protein concentration and activity in both laboratory and field conditions. Similarly, the coral *A. milepora* and the calcareous algae *Halimeda opuntia* exposed to the herbicide diuron (0–30 μg/L) had this enzyme activity enhanced (in preparation). On the other hand, some studies also evaluated the effects of environmental pollution on carbonic anhydrase in terms of gene expression; for example, Balbi et al. (2017) [104] showed that the mussel *M. galloprovincialis* exposed for 48 h to polystyrene nanoplastics had a down-regulation of about 40% in this gene transcription. Similarly, Capolupo et al. (2018) [105] demonstrated that the same species also experienced a reduction in carbonic anhydrase gene expression following exposure to polystyrene microplastics for 48 h.

At this point, it is clear that carbonic anhydrase responds to environmental pollution in a very predictable way, both in terms of gene transcription and enzymatic activity. As it was demonstrated, a great diversity of pollutants can reduce this enzyme activity and/or gene transcription in many calcifying animals. Therefore, there is sufficient evidence to sustain the idea that carbonic anhydrase is a good biomarker of environmental pollution, despite confounding factors such as phylogeny, type of contaminant, exposure period, and/or concentration. As a matter of fact, this enzyme has already been used in the context biomonitoring studies. Interestingly, the pattern of response of carbonic anhydrase in ecotoxicological studies performed under field conditions was opposite to that observed in laboratory experiments; for example, Santos et al. (2017) [106] showed that this enzyme was enhanced in the oyster *Crassostrea rhizophorae* collected in a highly impacted zone (Paraíba Estuary, Paraíba state, Brazil), in comparison to an area of environmental preservation (Mamanguape Estuary, Paraíba state, Brazil). Similarly, Azevedo-Linhares and Freire (2015) [107] demonstrated that the same oyster species also showed elevated activity of this enzyme when animals from impacted zones were compared to control areas. Moreover, Caricato et al. (2010) [103] demonstrated in a translocation study that the mussel *M. galloprovincialis* displayed an elevation in the carbonic anhydrase activity when exposed for 30 days to a polluted site (Mar Grande of Taranto, Taranto, Italy) in comparison to a reference site (S. Maria of Leuca, Lecce, Italy). 

### 4.4. Combined Impacts: The Role of Interactions

As already discussed in this work, local and global impacts are easily distinguishable and can be individually studied under laboratory conditions. However, these impacts occur together and interactive effects are expected. For example, Ivanina et al. (2013) [97] demonstrated that the marine bivalves *C. virginica* and *Mercenaria mercenaria* exposed to heat stress (5 °C temperature elevation) under acidified water experienced an increase in carbonic anhydrase activity in mantle tissue. Similarly, Fonseca et al. (2017) [52] showed that the coral *M. harttii* exposed to copper (3.8–8.6 μg/L) in elevated temperatures (temperature elevation of 1.6 and 2.3 °C) for 4 and 8 days had elevated carbonic anhydrase activity. Nonetheless, 12 days were sufficient for animals to recover and differences were no longer observed. In accordance, Bielmyer-Fraser et al. (2018) [108] demonstrated that the coral *Acropora cervicornis* exposed to 20 μg/L copper and water acidification (7.67 pH) for 4 days showed enhancement in this enzyme activity; however, this result was not observed in the coral *P. damicornis*. Moreover, combined effects were also showed for the coral *M. harttii*, that is, when intermediate water acidification (pH 7.80) was combined with many copper exposure concentrations (1.6, 2.3, and 3.2 μg/L) for 35 days, observed combined effects were mostly additive or synergistic. Similarly, when a low level of copper (1.6 μg/L) was combined with many degrees of acidification (pH levels of 7.80, 7.50, and 7.20), all observed interactions were also additive or synergistic. In all the cases, carbonic anhydrase activity was reduced [83]. Interestingly, Marangoni et al. (2019) [83] also showed that when M. harttii was exposed to 2.3 and 3.2 μg/L copper under more intense water acidification (7.50 and 7.20 pH) for 35 days, interactions were mostly antagonistic. Similarly, the combination of copper exposure (1.6; 2.3; and 3.2 μg/L) and ocean acidification (7.80, 7.50, and 7.20 pH) for 15 days also induced mostly antagonistic effects. Moreover, Moreira et al. (2016) [93] showed that the oyster species *C. angulata* and *C. gigas* displayed inhibition of this enzyme following exposure to a low pH level (pH 7.30) combined with arsenic (2.79 mg/L) over 4 weeks. Finally, Kaniewska et al. (2015) [109] demonstrated that the coral *A. millepora* exposed to an elevated temperature (28 °C) and acidified water (pH 7.68) for 5 weeks had down-regulated carbonic anhydrase genes.

## 5. Corals in the Spotlight of Biomonitoring Programs: Combining Carbonic Anhydrase Assessment with Specific Organismal Analyses

As a ubiquitous enzyme, carbonic anhydrase can be used as a biomarker in a wide range of organisms. However, we argue that distinct organismal biomarkers that can be assessed in calcifying animals, especially in corals, represent an additional dimension that, if explored in combination with carbonic anhydrase evaluation, can greatly enhance the quality of biomonitoring programs and environmental studies. This statement is based on the notion that multi-level biomarker assessment can be greatly beneficial to this kind of study. Taking into account the role of carbonic anhydrase on the supply of inorganic carbon to both calcification and photosynthesis [27], researchers can evaluate endpoints related to these processes as organismal biomarkers [83,110,111]. Beyond that, we argue that corals possess unique characteristics that can be considered to be particularly interesting for bioindicator species, considering that these animals are usually found in the form of large sessile colonies that can be tracked and re-assessed over time. In this way, biomarkers can be assessed in the same individuals, reducing analytical bias related to intraspecific variations, such as genetic diversity. Moreover, the same colonies can be evaluated over decades, as these animals are long-living organisms [112].

There are several methodologies to assess calcification in coral species. Under laboratory experiments, this process can be evaluated using the buoyant weight technique [113], which consists of weighting the coral in a known water volume and estimating the skeleton weight from this measure. Similarly, the calcification rate can also be assessed using the alkalinity anomaly technique, which is based upon the indirect assessment of CaCO_3_ consumption by the corals [114,115]. The interesting aspect of these two methodologies is that animals remain alive after the process; therefore, the same individual can be evaluated many times. However, as animals have to be weighed or maintained in an incubation chamber, these analyses are not suitable for field studies. Nonetheless, there are other methodologies that can be used in this context. One of the most common approaches is the assessment of annual density bands, which can be observed in coral slices drilled across the longitudinal or vertical growth axis of a coral core [116,117,118,119]. In this way, it is possible to assess two major variables that are used to evaluate calcification patterns. The first of them is the linear extension rate, which is how much coral growth can be observed between years. The second characteristic is the skeletal density, which can be measured via the direct quantification of weight and volume, or by scanning methodologies such as X-ray, computerized tomography, or gamma densitometry [116]. From these two parameters, it is also possible to derive the annual calcification rate, which is the mass of CaCO_3_ deposited by the coral in the form of a calcified skeleton per year [116].

Coral bleaching is the term for the loss of the mutualistic relationship between corals and their photosynthetic intracellular endosymbionts, zooxanthellae from the genus *Symbiodinium*, caused by anthropogenic impacts [120]. With the disruption in this relationship, endosymbionts are expelled from the host cells, leading to translucid tissues that expose the brilliant-white aragonite skeleton that lies beneath [121]. As this relationship can provide for up to 90% of the corals energetic demand [122], its breakdown can be greatly detrimental to these animals [121,122]. As manner of fact, it is already known that bleached corals grow and reproduce less and are more likely to die [123]. Taking into account all the above-mentioned information, coral bleaching has already been indicated as a potent organismal biomarker to track coral’s health status [124]. Interestingly, this endpoint can be easily assessed both under laboratory and field conditions by visual comparation with reference cards in a very reliable and inexpensive way [111].

## 6. Conclusions

It is demonstrated in this review that carbonic anhydrase activity assessed in calcifying animals responds to lowered water pH with a remarkable reproducibility, being reduced in the great majority of the studies. It is important to note that this result was observed regardless of phylogeny (evolutionary relatedness between species), acidification intensity, and the amount of time animals were exposed to acidified water. Similar results were also observed in terms of gene transcription, but in this case, a few studies showed that carbonic anhydrase gene transcription can also be raised by calcifying animals following exposure to acidified water.

The impact of water temperature elevation was also reviewed in this study and it is demonstrated that heat stress elevates the carbonic anhydrase activity in calcifying animals, but there are a reduced number of available studies that have evaluated this. Also, this review shows that an elevated temperature has mixed effects in terms of carbonic anhydrase gene transcription, that is, in some cases, these genes were up-regulated in calcifying animals and in other cases these genes were down-regulated in these species.

This work also reviewed the impacts of environmental pollution in carbonic anhydrase. It is clear that, under laboratory conditions, this enzyme activity and gene transcription are inhibited in calcifying organisms exposed to a multitude of contaminants, both organic and inorganic, regardless of phylogeny, exposure period, and contaminant concentration. Conversely, a few studies performed under field conditions showed that two bivalve mollusks actually raised the carbonic anhydrase activity in polluted areas.

Finally, this review showed that carbonic anhydrase is also very responsive when environmental stresses are evaluated in combination. Interestingly, it is observed that when heat stress is part of this association, this enzyme activity is elevated in calcifying animals, either when combined with water acidification or contamination. On the other hand, if the combined stressors are lowered pH and contaminants, carbonic anhydrase activity is suppressed.

In consideration of the above, it is possible to conclude that all dimensions in which carbonic anhydrase can be evaluated—that is, gene transcription, protein concentration, and enzymatic activity—are very responsive to heat stress, water acidification, and environmental contamination, and can be used as molecular and biochemical biomarkers in biomonitoring programs using calcifying animals as bioindicator species. In this way, this enzyme provides a potent biological tool for the assessment of global and local impacts and possible interactions between them. Also, within this context, these analyses can be used as predictive endpoints of populational and ecosystem damages. Finally, it is suggested that, when applicable, calcification and bleaching should be used as organismal biomarkers in association with carbonic anhydrase evaluation to assess the current health status of colonies and to track on-going impacts at the population level.
